# An inexpensive open source 3D-printed membrane feeder for human malaria transmission studies

**DOI:** 10.1186/s12936-018-2436-9

**Published:** 2018-08-03

**Authors:** Kathrin Witmer, Ellie Sherrard-Smith, Ursula Straschil, Mark Tunnicliff, Jake Baum, Michael Delves

**Affiliations:** 1 Department of Life Sciences, Imperial College, London, SW7 2AZ UK; 2 MRC Centre for Global Infectious Disease Analysing, Department of Infectious Disease Epidemiology, Imperial College, London, W2 1PG UK

**Keywords:** Malaria, Transmission, Gametocyte, Mosquito, SMFA

## Abstract

**Background:**

The study of malaria transmission requires the experimental infection of mosquitoes with *Plasmodium* gametocytes. In the laboratory, this is achieved using artificial membrane feeding apparatus that simulate body temperature and skin of the host, and so permit mosquito feeding on reconstituted gametocyte-containing blood. Membrane feeders either use electric heating elements or complex glass chambers to warm the infected blood; both of which are expensive to purchase and can only be sourced from a handful of specialized companies. Presented and tested here is a membrane feeder that can be inexpensively printed using 3D-printing technology.

**Results:**

Using the *Plasmodium falciparum* laboratory strain NF54, three independent standard membrane feeding assays (SMFAs) were performed comparing the 3D-printed feeder against a commercial glass feeder. Exflagellation rates did not differ between the two feeders. Furthermore, no statistically significant difference was found in the oocyst load nor oocyst intensity of *Anopheles stephensi* mosquitoes (mean oocyst range 1.3–6.2 per mosquito; infection prevalence range 41–79%).

**Conclusions:**

Open source provision of the design files of the 3D-printed feeder will facilitate a wider range of laboratories to perform SMFAs in laboratory and field settings, and enable them to freely customize the design to their own requirements.

**Electronic supplementary material:**

The online version of this article (10.1186/s12936-018-2436-9) contains supplementary material, which is available to authorized users.

## Background

Transmission of malaria from vertebrate host to mosquito is mediated by the mature sexual stages of the *Plasmodium* life cycle—male and female gametocytes. Gametocytes sense their uptake into the mosquito midgut by a decrease in temperature and the presence of mosquito-derived xanthurenic acid and rapidly differentiate into male and female gametes [1]. Gametes fuse and fertilization ensues, with the resultant motile ookinetes migrating to and through the midgut epithelium, where they develop into oocysts upon contacting the basal lamina. Artificial feeding of mosquitoes using gametocyte-infected blood in a membrane feeding system is a mainstay of *Plasmodium* transmission stage research to study cell biology, vaccine and anti-malarial drug development [2–4]. At its simplest, membrane feeding requires a gametocyte-containing blood meal, a source of heat to maintain the blood at 37 °C (to ensure gametocytes are not prematurely activated and to simulate body temperature to promote mosquito feeding) and a membrane around the blood to simulate the skin of the host [5]. Two solutions are commonly employed to perform the Standard Membrane Feeding Assay (SMFA): (1) Hemotek^®^ [6], in which infected blood is placed between electrically heated feeder reservoirs and a surrounding membrane. (2) Water-jacketed glass or plastic feeders in which heated water from a circulating water bath passes through the feeder and warms the infected blood sample surrounded with a membrane [7]. This type of glass feeder is most commonly applied in field settings [8] using a standardized protocol [5]. Whilst both are effective, they can be expensive for laboratories with limited resources and can only be obtained from a few suppliers, thus limiting their availability.

3D-printing refers to any process using computer control to create a three-dimensional object. The 3D-printing revolution has opened up professional design and manufacture on a small-scale to mainstream users, enabling rapid transitions from an initial design to the finished product. Presented here is a simple two-piece water-jacketed membrane feeder designed to hold a volume of 500 µl. The design for the feeder used here is supplied in OBJ format (Additional files 1 and 2), which can be opened in any computer-aided design (CAD) package for 3D-printing—many of which are provided for free download on the Internet. Using the files presented here, the feeder can be 3D-printed directly and inexpensively by stereolithography by any equipped lab or commercial 3D-printing provider. Alternatively, by using a CAD package the size of the feeder can be up- or downscaled to hold more or less volume respectively.

This study validates and compares the acrylic resin 3D-printed feeder to a conventional glass feeder. Exflagellation rates as well as oocyst counts indicate that there is no significant difference between the two, within the statistical power given by triplicate SMFAs used as standard by the research community. The design of the feeder is provided here, enabling others to gain inexpensive access to equipment needed to perform SMFAs and making future modifications or improvements to the design straightforward.

## Methods

### Design and production of the membrane feeder

The design for the membrane feeder was modelled in the free open source CAD modeller Art of Illusion v3.0.2 (http://www.artofillusion.org/) using combined Boolean modelling of simple geometric shapes. The 3D models of the top and bottom halves of the feeder were commercially printed in USP VI medical-grade “Fine Detail Plastic” acrylic resin (VisiJet M3 Crystal) using stereolithography by Shapeways (https://www.shapeways.com/). The 3D modelling files are freely available for download and modification (Additional files 1, 2) under a Creative Commons 4.0 Attribution International license. Before first use, both halves of the feeder were washed extensively with tap water to remove any unpolymerized resin and allowed to dry before being glued together with cyanoacrylate cement (“superglue”).

### *Plasmodium falciparum* standard membrane feeding assay (SMFA)

*Plasmodium falciparum* NF54 gametocytes were prepared by standard methods [7] and between 13–16 days after culture induction were fed to 3–7 days old *Anopheles stephensi* mosquitoes. All culture manipulations were performed at 37 °C to prevent premature activation of the gametocytes. Briefly, 200–300 μl of pre-warmed fresh human red blood cells (RBC) (O+ male) were added at the bottom of a 15 ml conical tube, and the gametocyte culture added on top. The mixture was pelleted using a heated centrifuge at 500 rcf for 5 min at 38 °C. The supernatant was removed with an aspirator and the RBC/gametocyte pellet was mixed with pre-warmed human serum in a ratio of 2:3. The conventional glass feeder (Dixon Glass) and the 3D-printed feeder were attached to a 38 °C circulating water bath (Grant Instruments) chained in a loop with silicone tubing (total circulation loop = ~ 50 cm). The bottom of the pre-warmed feeders was covered with Parafilm^®^ stretched in both directions to make a thin membrane. The RBC/gametocyte/serum mixture was equally distributed into both feeders and mosquitoes were allowed to feed for 20–30 min at ambient room temperature of 21 °C.

After mosquito feeding, the gametocyte/RBC/serum mixture was removed into prewarmed microcentrifuge tubes and put on a heating block at 37 °C to test for exflagellation (see below). The feeder set up was disassembled and feeders were washed with hot water. Exposure to 10% bleach for 7 consecutive days did not affect the mechanical properties or durability of the 3D-printed feeder and permits a more rigorous decontamination procedure if required for safety reasons such as SMFAs with unscreened blood.

After infection, mosquitoes were maintained at 26 °C and 80% humidity. 24 h post-infection, unfed mosquitoes were removed. After 9 days, mosquito midguts were removed and stained in 0.1% mercurochrome in PBS for 15 min. Oocysts per midgut were counted at 20× magnification using a light microscope.

### Post-feed exflagellation assay

After membrane feeding and whilst the feeders were still assembled and warm, the remaining RBC/gametocyte/serum mixture was removed into a microcentrifuge tube and kept at 37 °C (see above). 10 µl of the RBC/gametocyte/serum mixture was then added to 90 µl incomplete ookinete medium (100 µM xanthurenic acid, 2 g/l sodium bicarbonate, 50 mg/l hypoxanthine in RPMI1640-HEPES, pH 7.4) [9], transferred to a FastRead Counting Slide and incubated at room temperature. After 10–15 min, exflagellation centres were counted using brightfield microscopy with a 10× objective three times for each feeder. This number was then multiplied by ten to express exflagellation per ml of feeder.

### Statistical analysis

Statistical analyses for exflagellation rates and oocyst numbers were performed using GraphPad Prism version 7 or R version 3.5. Exflagellation was compared using paired t-test; oocyst intensity and prevalence were compared using a zero-inflated Poisson regression to accommodate the distribution of the count data [10]. In addition, a Bayesian approach was used to demonstrate there is no statistical difference between experimental replicates. Zero-inflated Poisson probability distributions were fitted using Hamiltonian Monte Carlo sampling methods [11]. The probability function is:

$$p\left( {y_{n} |\theta ,\lambda } \right) = \left\{ {\begin{array}{*{20}l} {\theta + \left( {1 - \theta } \right) \times Poisson\left( {0|\lambda } \right)} \\ {\left( {1 - \theta } \right) \times Poisson\left( {y_{n} |\lambda } \right)} \\ \end{array} } \right.$$

where *y* represents the number of oocysts observed in *n* mosquitoes, and there is a probability θ of drawing a zero and a probability 1 − θ of drawing from a Poisson distribution with mean parameter λ. Four chains were initialised to assess the convergence of 2000 iterations, the first 1000 of each were discarded as burn in. The posterior distributions of parameters (4000 iterations) and 95% Bayesian credible intervals were estimated, posterior checks were performed using shinystan library version 2.5 and visually confirmed to fit the data (Fig. 3). Power calculations of feed data were estimated in R using the *pwr* library.

## Results

### Assembly and operation of the membrane feeder

**Fig. 1:**
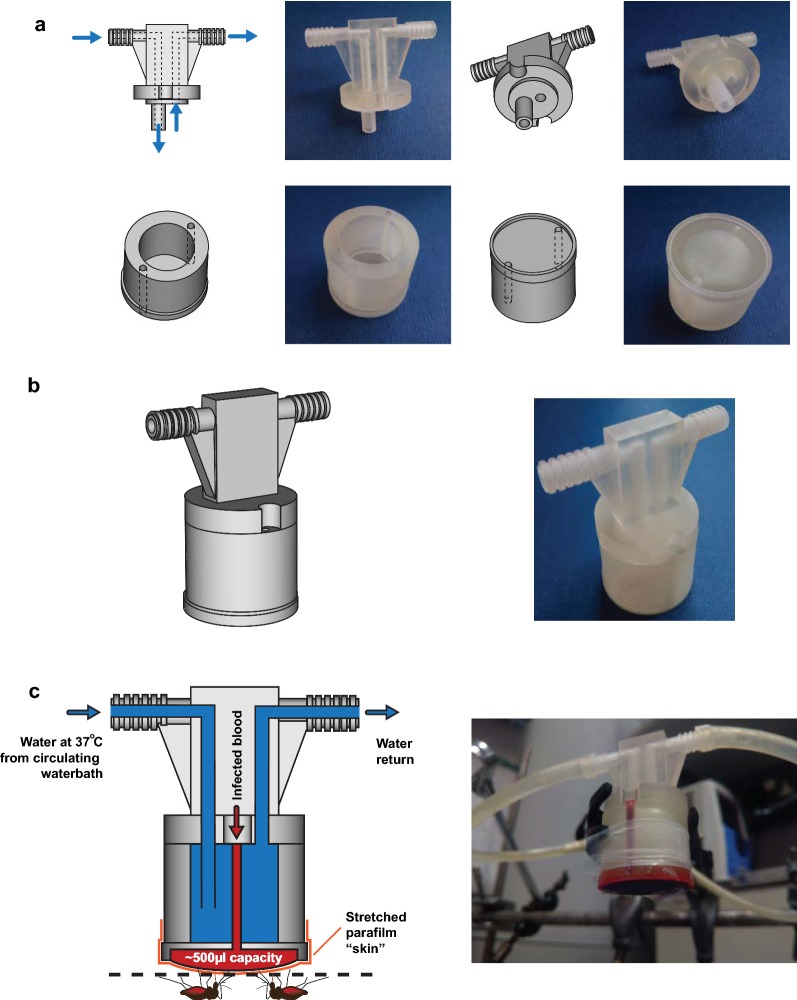
Assembly and operation of the 3D-printed membrane feeder. **a** The membrane feeder was designed in two parts, a top chamber that connects to a circulating water bath and a bottom chamber holding a water reservoir and the RBC/gametocyte/serum sample on the underside. **b** Both pieces are glued together into a single, watertight unit. **c** When in operation, circulating warm water maintains the temperature of the gametocyte-infected blood sample that is injected through access holes and sits between a layer of stretched Parafilm^®^ and the underside of the feeder

The 3D-printed membrane feeder was designed and manufactured in two parts (Additional files 1, 2)—a bottom chamber to accommodate the infected blood sample and circulating heated water, and a top chamber to accommodate connection to the circulating water source (Fig. 1a). To assemble the feeder, the two parts were glued together with cyanoacrylate cement (“superglue”) so that the notches in the top part align with the two injection holes in the bottom part (Fig. 1b). To operate, the feeder was connected to a circulating water bath at 38 °C (to ensure a continual supply of water to warm the feeder) and allowed to equilibrate for 20 min. A piece of Parafilm^®^ stretched thin in both directions was wrapped over the underside of the feeder and a 500 µl sample containing RBC/gametocytes/serum was introduced via the injection holes (Fig. 1c).

## 3D-printed membrane feeder supports the transmission of *P. falciparum* to mosquitoes

**Fig. 2:**
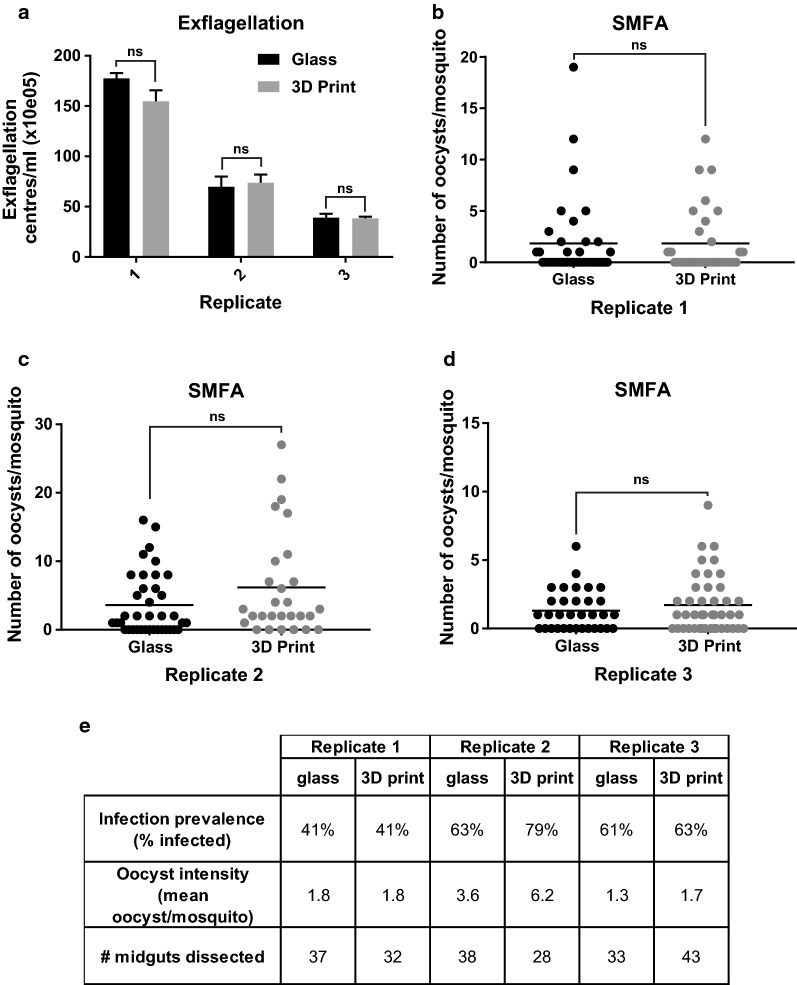
Comparative *P. falciparum* SMFAs with a commercial glass feeder and 3D-printed feeder. **a** After feeding, blood was sampled from the feeders and exflagellation was induced and quantified. Exflagellation is indicated per millilitre per feeder. Three independent biological replicates are shown (1–3). No statistically significant difference was found. **b**–**d** Three standard membrane feeding assays (SMFAs) were performed in which the RBC/gametocyte/serum sample was split between the two feeders, mosquitoes allowed to feed and midgut oocysts quantified 9 days later. Each dot represents one mosquito midgut. The mean oocyst number is indicated with a straight horizontal line. No statistically significant difference was found between the two feeders. **e** Infection parameters quantified from SMFA replicates 1–3

**Fig. 3:**
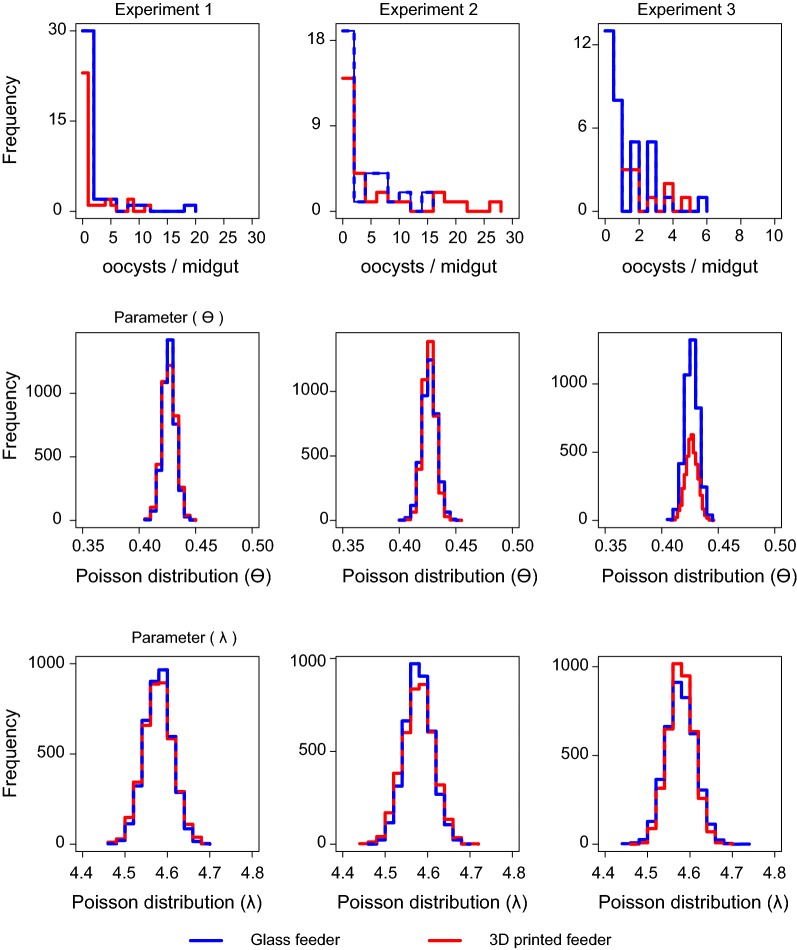
Statistical analysis of SMFA data. **a** Raw data for the number of oocysts per fed mosquito. These oocyst intensities were compared using a zero-inflated Poisson regression to accommodate the distribution of the count data. A Bayesian approach was used to demonstrate there is no statistical difference between experimental replicates. Zero-inflated Poisson probability distributions were fitted using Hamiltonian Monte Carlo sampling methods described in the main manuscript. There is a probability *θ* of drawing a zero and a probability (1 − *θ*) of drawing from a Poisson distribution with mean parameter *λ*. Four chains were initialised to assess the convergence of 2000 iterations, the first 1000 were discarded as burn in. The posterior distributions of parameters (4000 iterations) **b** *θ* and **c** *λ* for each experimental replicate (columns 1–3 respectively) are shown, posterior checks demonstrate the oocyst counts are not statistically different when mosquitoes feed on a glass (blue) or a 3D-printed (red) standard membrane feeding assay (SMFA)

The feeder was tested head-to-head against a commercial glass membrane feeder. The RBC/gametocyte/serum mixture was divided equally between the two. Separate pots of *An. stephensi* mosquitoes were allowed to feed on both feeders and residual blood was collected to assess male gametocyte exflagellation levels by quantification in a haemocytometer. With three independent biological replicates derived from different gametocyte cultures and different mosquito generations, both the glass and 3D-printed feeders showed no significant differences in exflagellation post-feeding (paired t test; p = 0.26, 0.23 and 0.88 respectively for replicates 1–3) (Fig. 2a). This suggests both that the heat transfer from the circulating water is sufficient to preserve gametocyte viability and that the acrylic resin photopolymer material of the 3D-printed feeder is non-toxic and does not affect the parasites during feeding (Fig. 2a). Nine days later when *An. stephensi* oocyst burden was assessed (Fig. 2b), it was found that there was no significant difference in oocyst intensity (Zero-inflated regression (Binomial with logit link): p = 0.994, 0.188 and 0.756, respectively for replicates 1–3). The Bayesian analysis showed clearly that in each experimental replicate of either membrane feeder, there is no difference in the data distribution or the parameter estimates that can describe these data. Even with 4000 posterior estimates for the parameters θ and λ, no difference was observed in the range of estimates for any of the experimental replicates (Fig. 3). The infection prevalence of *An. stephensi* mosquitoes was not statistically different between each feeder in all three biological replicates (Fisher’s Exact test; p ≥ 0.99, 0.28, > 0.99, respectively for replicates 1–3) (Fig. 2b). Assuming a statistical power of 0.8, a difference of > 65% in prevalence between the two feeders in all three replicates would be significant with 95% confidence.

## Discussion

Under standard SMFA conditions, the 3D-printed, acrylic resin photopolymer membrane feeder was found to give equally successful *P. falciparum* infections in *An. stephensi* mosquitoes when compared to a glass counterpart but at a third of the cost (€20 versus €58).

Like glass feeders, the printed unit is robust and reusable, providing a cost-effective alternative to existing solutions. The only operational difference found in testing of the 3D printed feeder compared to conventional protocols was that it required extended washing in tap water after feeding experiments to remove residual blood due to the rough unfinished surface of the acrylic resin. This could be mitigated in the future by polishing the surface of the feeder or printing at higher resolution. Furthermore, the 3D-printed feeder was fully functional even after a consecutive 7 day exposure to 10% bleach solution—frequently used to decontaminate feeders in field-based experiments where unscreened blood is used. A final operational consideration required to be optimised by the user is water bath temperature that may need to be decreased in tropical areas with high ambient temperatures to prevent heat damage to the gametocytes.

## Conclusions

The 3D-printed feeder design enables researchers to inexpensively produce their own SMFA feeders as an alternative to expensive and fragile glass feeders that require specialist manufacturing. This new 3D-printed feeder can be used in a wide range of applications in addition to standard SMFAs, as it is not limited to the species used here. Application might include the assessment of vector competence for malaria [12], the epidemiological assessment of the infectious reservoir for malaria [13], clinical drug trials [14], and transmission-blocking studies [15, 16].

## Additional files

**Additional file 1.** Feeder bottom. 3D CAD file of the bottom part of the membrane feeder.

**Additional file 2** Feeder top. 3D CAD file of the top part of the membrane feeder.

## Abbreviations

RBC: red blood cell
SMFA: standard membrane feeding assay
